# Adherence to Mediterranean Diet among Lebanese University Students

**DOI:** 10.3390/nu13041264

**Published:** 2021-04-12

**Authors:** Joanne Karam, Maria del Mar Bibiloni, Mireille Serhan, Josep A. Tur

**Affiliations:** 1 Research Group on Community Nutrition and Oxidative Stress, University of Balearic Islands-IUNICS, IDISBA & CIBEROBN (Physiopathology of Obesity and Nutrition CB12/03/30038), E-07122 Palma de Mallorca, Spain; joannemkaram@gmail.com (J.K.); mar.bibiloni@uib.es (M.d.M.B.); 2 Nutrition & Dietetics Department, Modern University for Business & Science, Damour 5660, Lebanon; 3 Faculty of Health Sciences, University of Balamand, Balamand Al Kurah 3843, Lebanon; mireille.serhan@balamand.edu.lb

**Keywords:** Mediterranean diet, dietary habits, university students, Lebanon

## Abstract

Scarce studies described eating habits and diet quality among university students in Lebanon. The aim of this study is to assess the rate of adherence to the Mediterranean diet (MedDiet) among Lebanese university students. A cross-sectional nutritional survey was carried out on 525 students (53% men, 18–25 years old) from the University of Balamand, Lebanon. Adherence to the MedDiet was assessed using a validated 14-item MedDiet adherence score. Mean adherence to the MedDiet was 7.96 (standard deviation 2.2), and it was adequate in 59% of participants. Adherence to the MedDiet was higher in older students and nonsmokers. Legumes, vegetables, fruits, and nuts were consumed according to the MedDiet standards among a minimum of 48.4% and a maximum of 69.5% of participants. Chicken, turkey, or rabbit was preferred by 66.9% of participants instead of beef, pork, hamburgers, or sausages; however, just 56.2% of participants showed adequate intake of red meat, hamburger, or meat products. Only 28.8% of them referred to an adequate intake of fish or shellfish. Most of the participants (86.3%) used olive oil as the main added fat, and 67.2% reported a low intake of butter and derivatives. Sofrito was also very usual among participants (79.6%). Only half of the studied sample reported an adequate intake of sweet or carbonated beverages and commercial sweets or pastries. Among the assessed sample, half the participants showed adequate adherence to the MedDiet; however, the mean of adherence among the sample is low.

## 1. Introduction

In Lebanon, the prevalence of overweight and obesity has attained alarming rates in adults, adolescents, and children due to an energy-dense diet and lack of physical activity [1]. According to a systematic review, high adherence to the Mediterranean diet (MedDiet) was significantly related to less overweight and more weight loss [2]. The benefits of the MedDiet may be due to the synergistic combination of a balanced ratio of n-6 and n-3 essential fatty acids, high amounts of dietary fibers, high oleic acid, and polyphenols consumption [3]. Non-nutritional aspects, linked in one way or another to food consumption, have been suggested to contribute to the beneficial effect of the MedDiet; these include physical activity, consumption of fresh and local products, and eating in groups. It is important to assess the degree of adherence to the MedDiet through accurate measurement tools such as dietary scores [4]. Poor adherence to MedDiet has been found among the Lebanese adult population [5]. Directing governments’ political actions toward spreading adherence to the MedDiet’s principles as much as possible among the population could help to tackle the obesity epidemic [6]. 

The university phase may be the first phase of life when most teenagers start making their own food choices along with other choices. Dietary habits of young adults are affected by the fast-food market; this is reflected by increased overweight and obesity among this age group [7]. However, universities may provide an ideal forum for reaching out to many young adults through nutrition education programs that may positively influence students’ eating habits [7]. 

Scarce studies described eating habits among university students in Lebanon [7]. No study was found assessing adherence to MedDiet among this age group. Health in old age is related to health in earlier years of life [8]. The aim of this study was to assess the adherence to MedDiet among Lebanese university students. 

## 2. Methods 

### 2.1. Study Population

This is a cross-sectional study carried out 8 on 525 students (53% men) from three different campuses of the University of Balamand in Lebanon (Faculty of Health Sciences, Beirut; ALBA, Sin el Fil and Main campus, Koura). They were asked to answer by themselves a questionnaire in the presence of the interviewer to help in any clarification needed. The study was conducted according to the guidelines laid down in the Declaration of Helsinki, and all procedures were approved by the Balamand University Ethics Committee (approval reference 1.2019). Written informed consent was obtained from all participants.

### 2.2. Assessment of Adherence to the Mediterranean Diet

The questionnaire was divided into two parts—non-nutritional general information (sex, age, working status, smoking status, willingness to change diet) and nutritional information made by measurement of the adherence to MedDiet (assessed using a 14-item MedDiet adherence score, developed by the Prevención con Dieta Mediterránea (PREDIMED) consortium for immediate feedback [9,10]). The score of adherence was calculated by summing the points of the 14 questions (“Yes” answer was one point, “No” was zero points; the resulting score ranged from 0 to 14). The higher the score was, the greater the adherence to the Mediterranean diet. This questionnaire has been adapted to and validated for the Spanish population [11] and then adapted in an English version to be implemented in other populations [12]. This questionnaire has been used also in samples of young adults [13].

### 2.3. Statistics

Statistics were performed using the SPSS statistical software package version 25.0 (SPSS Inc., Chicago, IL, USA). Data were tested for normality using the nonparametric test Kolmogorov–Smirnov test on SPSS. Results are shown as mean and standard deviation (SD) for normally distributed data, and median and interquartile range (IQR) in the case of non-normal distribution. Nonparametric tests (chi-square test, Mann–Whitney U-test, or Kruskal–Wallis test) were used when the sample was not normally distributed. Results were considered significant if *p*-value < 0.05.

## 3. Results

The mean MedDiet adherence score was 8.0 (±SD 2.2) with no difference between genders (Table 1). MedDiet adherence score was higher the older the student was (18–21 years old: 7.8 ± 2.2; 22–25 years old: 8.2 ± 2.0). On non-nutritional background, students who worked in parallel showed higher results of MedDiet adherence score (8.4 ± 2.1) than those that only studied (7.9 ± 2.2), and nonsmokers (8.1 ± 2.1) also showed higher scores than smokers (7.7 ± 2.3). Higher adherence was found among students with a willingness to change diet.

Table 2 shows stratification of adherence to MedDiet items. On nutritional background, legumes, vegetables, fruits, and nuts were consumed according to the MedDiet standards among a minimum of 48.4% and a maximum of 69.5% of participants. Chicken, turkey, or rabbit was preferred by 66.9% of participants instead of beef, pork, hamburgers, or sausages; however, just 56.2% of participants showed adequate intake of red meat, hamburger, or meat products. Furthermore, only 28.8% of them referred to an adequate intake of fish or shellfish. Most of the participants (86.3%) used olive oil as the main added fat, and 67.2% reported a low intake of butter and derivatives. Consumption of sofrito, which is a sauce made with olive oil, onion, garlic, and tomato, was also very usual among participants (79.6%). Only half of the studied sample reported an adequate intake of sweet or carbonated beverages (51.8%) and commercial sweets or pastries (not homemade) (50.7%). Men consumed more fish, nuts, and chicken, turkey, or rabbit than women; and women consumed more vegetables and sofrito than men.

Table 3 shows adequacy of adherence to MedDiet according to different variables. An adequate MedDiet adherence score (≥9) was observed in 59% of the participants. The highest percentage of inadequate MedDiet adherence score was found among students that are only devoted to their study. Other items showed a similar percentage of adherence to the adequacy of the MedDiet.

## 4. Discussion

The mean adherence to the MedDiet among Lebanese students was discrete (7.96 out of 14) but lower than adequate adherence score (≥9: healthy score to prevent cardiovascular diseases (CVD)) even though half of the studied population showed adequate adherence to the MedDiet [14,15]. No difference was observed among genders, unlike other studies among Lebanese university students showing a higher adherence to Lebanese MedDiet among women [16] and higher consumption of westernized diet among men [17]. 

The 14-point questionnaire to assess the adherence to the MedDiet was used by the PREDIMED group for immediate feedback in Spain [9,10], UK [11], and Greece [13]; it was also used as well to assess people at high risk of CVD, in 18–75-year-old German women [18] and in heart or lung transplant recipients aged ≥16 years old in Manchester [19]. The mean MedDiet adherence score estimated among those in high risk of CVD patients (5.47) [9,10], heart or lung transplant recipients (6.6) [19], and current Lebanese students was higher than those observed in Lebanese adults (4.2) estimated on a 10-point scale based on above and below median levels of consumption [5].

Adherence to MedDiet was higher the older the student was, similarly to previous results obtained in other Mediterranean populations [20]. Men (7.99) had a very slightly higher and not different MedDiet adherence score than women (7.92), similar to a study previously conducted in <30-year-old Lebanese adults [5] and another study developed in Spain among nondiabetic and diabetic males and females [21]. 

When non-nutritional outcomes were assessed, current Lebanese nonsmokers showed higher mean MedDiet adherence score and higher adequate score than smokers, as shown by several previous studies smoking and a healthier diet are correlated and often occur together as part of overall lifestyle patterns [22]. Students primarily worried about their health (around 30% of participants) also showed better adherence, which is quite similar to other Mediterranean students, whose better adherence was associated with better self-rated health [23]. This should be taken into consideration in planning strategies to increase the level of adherence to the MedDiet for students to make them aware of the major role of dietary habits in health.

Stratification of nutritional outcomes of the questionnaire revealed a relatively high spread of usage of olive oil in cooking (86.3%) although only 50.3% consume more than four teaspoons per day. The percentage of participants consuming food according to the MedDiet standards was higher than 50%, except for wine and fish. The consumption of olive oil and fish should be more widespread among students. This stratification revealed slight differences in the eating habits between men and women, unlike another study conducted among university students in Lebanon [7].

Moreover, a higher percentage of participants in this study answered “Yes” to questions Q3 (vegetables), Q4 (fruits), Q9 (legumes), Q14 (cooking with olive oil, onion, garlic, and tomato) than a study on coronary artery disease study [22]. In both studies, less than 50% of participants showed an adequate intake of wine conforming to MedDiet standards, but fish and fish products’ intake differed greatly between both studies; moreover, 28.8% of university students answered “Yes,” compared to 59% in a coronary artery disease study [24]. The low consumption of fish confirms the outcome of previous studies showing a low consumption of fish among the adult population living in Beirut, Lebanon [5,25]. To cook with olive oil, onion, garlic, and tomato is very usual among Mediterranean people since they are the ingredients of a typical Mediterranean sauce, which is a key component of the MedDiet and strongly associated with reduced risk of CVD mainly due to its phenolic, polyphenols, and carotenoids contents [25]. Lebanese population showed an alarming increase in nutrition-related CVD risk factors [26]. This should alert in applying strategies at both individual and community level to promote healthy dietary and lifestyle habits. Accordingly, the high consumption of the Mediterranean diet, which has been shown to protect from cardiovascular diseases (CVD) prognosis, diabetes, mortality, cancer, obesity, grant favorable health status, better biochemical profile, influence positively the quality of life the academic performance, the muscular fitness and mental health [27,28,29,30,31,32,33,34,35,36,37,38,39,40,41], may be a good way to implement healthy dietary habits, which would be easier to implement if they are joined to good sensory characteristics [42] and within the context of a healthy MedDiet.

The analysis of the adherence to MedDiet according to non-nutritional outcomes showed that the worst scores were found in current Lebanese students with political problems, economy, and future as main worrying issues (59.4% of all worrying issues). Indeed, the Lebanese state is in bad conditions, plagued by several chronic problems that include a long-lasting political vacuum, a major burden of millions of refugees, and the ongoing revolution against the state since October 2019. Lebanon ranks 73rd in the world by nominal gross domestic product (GDP) per capita (USD 8778) [43]. Lebanon also showed a human development index (HDI) value of 0.730 for 2018, which put the country in the category 93 of 189 countries and territories [44]. Therefore, it is not surprising the expressed main worry among Lebanese students and those who showed lower adherence and interest in improving their dietary pattern. Health as the main worrying issue was declared by 28.6% of students, and 50% of those showed inadequate adherence to the MedDiet. Accordingly, most of the students declared a clear willingness to change diet, which could be understood to improve their health status. In all cases, improving the dietary pattern of the Lebanese population will positively contribute to increasing public health in this country and improve the national situation, as recommended by the World Health Organization who urged promoting lifestyles including healthy diets [45].

### Strengths and Limitations

MedDiet has been assessed in Lebanon, and it should be taken into consideration in planning strategies to increase the public health nutrition among the Lebanese young population. This study has also several limitations that must be acknowledged. First, the applied questionnaire of MedDiet adherence score was validated for the Spanish population, but it was not validated in Lebanese students yet before conducting this investigation; however, it has been adapted to an English version and used in other samples of young adults. A second limitation is that all nutritional data and most of the nutritional assessment methods presented here are self-reported. Finally, this is a cross-sectional study, and therefore, we acknowledge that we are not able to draw causal conclusions but only observations.

## 5. Conclusions

The mean adherence to the MedDiet among Lebanese students was discrete but lower than the adequate adherence score even though half of the studied population showed adequate adherence to the MedDiet. They declared food consumption according to the MedDiet standards but not in a widespread way. Awareness should be spread among university students to highlight the benefits of MedDiet and encourage its adherence.

## Figures and Tables

**Table 1:** Adherence to MedDiet and related variables.

	All				Men				Women			
	*n*	Mean (SD)	Median (IQR)	*P* *	*n*	Mean (SD)	Median (IQR)	*P* *	*n*	Mean (SD)	Median (IQR)	*P* *
Total sample	525	8.0 (2.2)	8.0 (7.0–9.5)		278	8.0 (2.2)	8.0 (7.0–10.0)		247	7.9 (2.1)	8.0 (7.0–9.0)	
Age												
18–21	335	7.8 (2.2)	8.0 (7.0–9.0)	0.033	161	7.8 (2.3)	8.0 (7.0–9.0)	0.220	174	7.8 (2.1)	8.0 (6.0–9.0)	0.073
22–25	190	8.2 (2.0)	8.0 (7.0–10.0)		117	8.2 (2.1)	8.0 (7.0–9.0)		73	8.3 (2.0)	8.0 (7.0–10.0)	
Working status												
University student	434	7.9 (2.2)	8.0 (7.0–9.0)	0.018	221	7.9 (2.2)	8.0 (7.0–9.0)	0.049	213	7.9 (2.1)	8.0 (7.0–9.0)	0.219
University student and worker	91	8.4 (2.1)	9.0 (7.0–10.0)		57	8.5 (2.1)	9.0 (7.0–10.0)		34	8.2 (2.0)	8.0 (8.0–10.0)	
Smoking habits												
Yes	176	7.7 (2.3)	8.0 (6.3–9.0)	0.043	120	7.7 (2.4)	8.0 (6.0–9.0)	0.078	56	7.7 (2.2)	8.0 (7.0–9.0)	0.216
No/did not smoke in the past five years	349	8.1 (2.1)	8.0 (7.0–10.0)		158	8.2 (2.1)	8.0 (7.0–10.0)		191	8.0 (2.1)	8.0 (7.0–10.0)	
Willingness to change diet												
Yes	438	8.0 (2.1)	8.0 (7.0–9.3)	0.049	218	8.0 (2.3)	8.0 (7.0–9.3)	0.914	220	8.1 (1.9)	8.0 (7.0–9.8)	<0.001
No	87	7.5 (2.4)	7.0 (6.0–10.0)		60	7.5 (2.1)	8.0 (6.0–8.0)		27	6.5 (2.7)	6.0 (4.0–8.0)	

Abbreviations: SD, standard deviation; IQR, interquartile range; MedDiet, Mediterranean diet. * Evaluated by Mann–Whitney U-test.

**Table 2:** Stratification of adherence to MedDiet items.

	All				Men				Women				
	Yes		No		Yes		No		Yes		No		
	*n*	%	*n*	%	*n*	%	*n*	%	*n*	%	*n*	%	*P* *
Q1: Olive oil for cooking	453	86.3	72	13.7	237	85.3	41	14.7	216	87.4	31	12.6	0.465
Q2: Quantity of olive oil per day	264	50.3	261	49.7	150	54.0	128	46.0	114	46.2	133	53.8	0.074
Q3: Vegetables portion per day	351	66.9	174	33.1	173	62.2	105	37.8	178	72.1	69	27.9	0.017
Q4: Fruits portion per day	281	53.5	244	46.5	155	55.8	123	44.2	126	51.0	121	49.0	0.277
Q5: Red meat per day	295	56.2	230	43.8	159	57.2	119	42.8	136	55.1	111	44.9	0.623
Q6: Butter/ margarine portion per day	353	67.2	172	32.8	185	66.5	93	33.5	168	68.0	79	32.0	0.720
Q7: Sugary drinks portion per day	272	51.8	253	48.2	137	49.3	141	50.7	135	54.7	112	45.3	0.219
Q8: Wine portion per week	103	19.6	422	80.4	63	22.7	215	77.3	40	16.2	207	83.8	0.063
Q9: Legumes portion per week	365	69.5	160	30.5	184	66.2	94	33.8	181	73.3	66	26.7	0.078
Q10: Fish and fish products portion per week	151	28.8	374	71.2	97	34.9	181	65.1	54	21.9	193	78.1	0.011
Q11: Processed deserts portion per week	266	50.7	259	49.3	143	51.4	135	48.6	123	49.8	124	50.2	0.707
Q12: Nuts portion per week	254	48.4	271	51.6	153	55.0	125	45.0	101	40.9	146	59.1	0.001
Q13: Consumption of chicken preferably over meat	351	66.9	174	33.1	173	62.2	105	37.8	178	72.1	69	27.9	0.017
Q 14: Cooking vegetables, pasta, rice with olive oil, onion, garlic and tomato per week	418	79.6	107	20.4	212	76.3	66	23.7	206	83.4	41	16.6	0.043

* Men vs. women by chi-square test. MedDiet: Mediterranean diet.

**Table 3:** Adequacy of adherence to MedDiet according to different variables.

	Score < 9		Score ≥ 9			Crude OR (95% CI)
	*n*	%	*n*	%	*P* *	
Total sample	310	59.0	215	41.0		
Sex						
Men	166	59.7	112	40.3	0.743	1.04 (0.73–1.49)
Women	144	58.3	103	41.7		
Age						
18–21	206	61.5	129	38.5	0.130	0.64(0.44–0.94)
22–25	104	54.7	86	45.3		
Working status						
University student	266	61.3	168	38.7	0.022	0.54 (0.33–0.89)
University student and worker	44	48.4	47	51.6		
Smoking habits						
Yes	113	64.2	63	35.8	0.088	0.69 (0.48–1.01)
No/did not smoke in the past five years	197	56.4	152	43.6		
Willingness to change diet						
Yes	255	58.2	183	41.8	0.386	1.91 (1.19–3.03)
No	55	63.2	32	36.8		

Abbreviations: OR, odds ratio; CI, confidence interval; MedDiet, Mediterranean diet. * Evaluated by chi-square test.

## Data Availability

There are restrictions on the availability of data for this trial, due to the signed consent agreements around data sharing, which only allow access to external re-searchers for studies following the project purposes. Requestors wishing to access the trial data used in this study can make a request to pep.tur@uib.es.

## References

[1] Issa C., Darmon N., Salameh P., Maillot M., Batal M., Lairon D. (2010). A Mediterranean diet pattern with low consumption of liquid sweets and refined cereals is negatively associated with adiposity in adults from rural Lebanon. Int. J. Obes..

[2] Buckland G., Bach A., Serra-Majem L. (2008). Obesity and the Mediterranean diet: A systematic review of observational and inter-vention studies. Obes. Rev..

[3] Donini L.M., Serra-Majem L., Bullo M., Gil A., Salas- Salvado J. (2015). The Mediterranean diet: Culture, health and science. Br. J. Nutr..

[4] Rumawas M.E., Dwyer J.T., McKeown N.M. (2009). The development of the Mediterranean-style dietary pattern score and its applica-tion to the American diet in the Framingham Offspring Cohort. J. Nutr..

[5] Farhat A.G., Jaalouk D., Francis S. (2016). Adherence to the Mediterranean diet in a Lebanese sample. Nutr. Food Sci..

[6] D’Innocenzo S., Biagi C., Lanari M. (2019). Obesity and the Mediterranean Diet: A Review of Evidence of the Role and Sustainability of the Mediterranean Diet. Nutrients.

[7] Yahia N., Achkar A., Abdallah A., Rizk S. (2008). Eating habits and obesity among Lebanese university students. Nutr. J..

[8] Banerjee A., Nikumb V., Thakur R. (2013). Health Problems Among the Elderly: A Cross-Sectional Study. Ann. Med. Health Sci. Res..

[9] Martínez-González M.A., García-Arellano A., Toledo E., Salas-Salvadó J., Buil-Cosiales P., Corella D., Covas M.I., Schröder H., Arós F., Gómez-Gracia E. (2012). A 14-Item Mediterranean Diet Assessment Tool and Obesity Indexes among High-Risk Subjects: The PREDIMED Trial. PLoS ONE.

[10] Bouzas C., Bibiloni M.D.M., Julibert A., Ruiz-Canela M., Salas-Salvadó J., Corella D., Zomeño M.D., Romaguera D., Vioque J., Alonso-Gómez Á.M. (2020). Adherence to the Mediterranean Lifestyle and Desired Body Weight Loss in a Mediterranean Adult Population with Overweight: A PREDIMED-Plus Study. Nutrients.

[11] Schröder H., Fitó M., Estruch R., Martínez-González M.A., Corella D., Salas-Salvadó J., Lamuela-Raventós R., Ros E., Salaverría I., Fiol M. (2011). A Short Screener Is Valid for Assessing Mediterranean Diet Adherence among Older Spanish Men and Women. J. Nutr..

[12] Papadaki A., Johnson L., Toumpakari Z., England C., Rai M., Toms S., Penfold C., Zazpe I., Martínez-González M.A., Feder G. (2018). Validation of the English Version of the 14-Item Mediterranean Diet Adherence Screener of the PREDIMED Study, in People at High Cardiovascular Risk in the UK. Nutrients.

[13] Bamia C., Martimianaki G., Kritikou M., Trichopoulou A. (2017). Indexes for Assessing Adherence to a Mediterranean Diet from Data Measured through Brief Questionnaires: Issues Raised from the Analysis of a Greek Population Study. Curr. Dev. Nutr..

[14] Patino-Alonso M.C., Recio-Rodríguez J.I., Belio J.F.M., Colominas-Garrido R., Lema-Bartolomé J., Arranz A.G., Agudo-Conde C., Gómez-Marcos M.A., García-Ortiz L. (2014). Factors Associated with Adherence to the Mediterranean Diet in the Adult Population. J. Acad. Nutr. Diet..

[15] Tobias-Ferrer J., Santasusana-Riera I., Cuadrench-Solórzano M., Gonzalez-Cabré M., Girbau-Tapias M., Sant-Masoliver C. (2015). Ad-herence to the Mediterranean diet in patients with coronary artery disease. Rev. Esp. Cardiol..

[16] Issa C., Jomaa L., Salamé J., Waked M., Barbour B., Zeidan N., Baldi I., Salameh P. (2014). Females are more adherent to Lebanese Med-iterranean Diet than males among university students. Asian Pac. J. Health Sci..

[17] Salameh P., Jomaa L., Issa C., Farhat G., Salamé J., Zeidan N., Baldi I. (2014). Assessment of dietary intake patterns and their correlates among university students in Lebanon. Front Public Health.

[18] Hebestreit K., Yahiaoui-Doktor M., Engel C., Vetter W., Siniatchkin M., Erickson N., Halle M., Kiechle M., Bischoff S.C. (2017). Valida-tion of the German version of the Mediterranean Diet Adherence Screener (MEDAS) questionnaire. BMC Cancer.

[19] Miura K., Entwistle T.R., Fildes J.E., Green A.C. (2017). Relative validity of short questionnaires to assess Mediterranean diet or low-fat diet adherence. J. Aging Res. Clin. Pract..

[20] Martínez E., Llull R., Bibiloni M.D.M., Pons A., Tur J.A. (2010). Adherence to the Mediterranean dietary pattern among Balearic Islands adolescents. Br. J. Nutr..

[21] Vidal-Peracho C., Tricás-Moreno J.M., Lucha-López A.C., Lucha-López M.O., Camuñas-Pescador A.C., Caverni-Muñoz A., Fanlo-Mazas P. (2017). Adherence to Mediterranean Diet pattern among Spanish adults attending a medical centre: Non-diabetic subjects and type 1 and 2 diabetic patients. J. Diabetes Res..

[22] Odegaard A.O., Koh W.P., Gross M.D., Yuan J.M., Pereira M.A. (2011). Combined lifestyle factors and cardiovascular disease mortality in Chinese men and women: The Singapore Chinese health study. Circulation.

[23] Barrios-Vicedo R., Navarrete-Muñoz E.M., García de la Hera M., González-Palacios S., Valera-Gran D., Checa-Sevilla J.F., Gimenez-Monzo D., Vioque J. (2015). A lower adherence to Mediterranean diet is associated with a poorer self-rated health in uni-versity population. Nut. Hosp..

[24] Nasreddine L., Hwalla N., Sibai A., Hamzé M., Parent-Massin D. (2006). Food consumption patterns in an adult urban population in Beirut, Lebanon. Public Health Nutr..

[25] Vallverdú-Queralt A., Rinaldi de Alvarenga J.F., Estruch R., Lamuela-Raventos R.M. (2013). Bioactive compounds present in the Med-iterranean sofrito. Food Chem. Food Chem..

[26] Nasreddine L., Naja F., Abla-Mehio S., Khalil H., Nada A., Nahla A. (2014). Trends in Nutritional Intakes and Nutrition-Related Car-diovascular Disease Risk Factors in Lebanon: The Need for Immediate Action. Lebanese Med. J..

[27] Koloverou E., Esposito K., Giugliano D., Panagiotakos D. (2014). The effect of Mediterranean diet on the development of type 2 diabetes mellitus: A meta-analysis of 10 prospective studies and 136,846 participants. Metabolism.

[28] Schwingshackl L., Missbach B., König J., Hoffmann G. (2015). Adherence to a Mediterranean diet and risk of diabetes: A systematic review and meta-analysis. Public Health Nutr..

[29] Li W.-Q., Park Y., Wu J.W., Goldstein A.M., Taylor P.R., Hollenbeck A.R., Freedman N.D., Abnet C.C. (2014). Index-based dietary patterns and risk of head and neck cancer in a large prospective study. Am. J. Clin. Nutr..

[30] Turati F., Trichopoulos D., Polesel J., Bravi F., Rossi M., Talamini R., Franceschi S., Montella M., Trichopoulou A., La Vecchia C. (2014). Mediterranean diet and hepatocellular carcinoma. J. Hepatol..

[31] Martinez-Gonzalez M.A., Bes-Rastrollo M., Serra-Majem L., Lairon D., Estruch R., Trichopoulou A. (2009). Mediterranean food pattern and the primary prevention of chronic disease: Recent developments. Nutr. Rev..

[32] Serra-Majem L., Roman B., Estruch R. (2006). Scientific Evidence of Interventions Using the Mediterranean Diet: A Systematic Review. Nutr. Rev..

[33] Sofi F., Cesari F., Abbate R., Gensini G.F., Casini A. (2008). Adherence to Mediterranean diet and health status: Meta-analysis. BMJ.

[34] Sanchez-Tainta A., Estruch R., Bullo M. (2008). Adherence to a Mediterranean type of diet and reduced prevalence of clustered car-diovascular risk factors in a cohort of 3204 high-risk patients. Eur. J. Cardiovasc Prev. Rehabil..

[35] Sofi F. (2009). The Mediterranean diet revisited: Evidence of its effectiveness grows. Curr. Opin. Cardiol..

[36] Dinu M., Pagliai G., Angelino D., Rosi A., Dall’Asta M., Bresciani L., Ferraris C., Guglielmetti M., Godos J., Del Bo’ C. (2020). Effects of Popular Diets on Anthropometric and Cardiometabolic Parameters: An Umbrella Review of Meta-Analyses of Randomized Controlled Trials. Adv. Nutr..

[37] Fernández-Medina I.M., Ruíz-Fernández M.D., Hernández-Padilla J.M., Granero-Molina J., Fernández-Sola C., Jiménez-Lasserrotte M.D.M., Lirola M.-J., Cortés-Rodríguez A.E., López-Rodríguez M.M. (2020). Adherence to the Mediterranean Diet and Self-efficacy as Mediators in the Mediation of Sleep Quality and Grades in Nursing Students. Nutrients.

[38] Cobo-Cuenca A.I., Garrido-Miguel M., Soriano-Cano A., Ferri-Morales A., Martínez-Vizcaíno V., Martín-Espinosa N.M. (2019). Adherence to the Mediterranean Diet and Its Association with Body Composition and Physical Fitness in Spanish University Students. Nutrients.

[39] Martín-Espinosa N.M., Garrido-Miguel M., Martínez-Vizcaíno V., González-García A., Redondo-Tébar A., Cobo-Cuenca A.I. (2020). The Mediating and Moderating Effects of Physical Fitness of the Relationship between Adherence to the Mediterranean Diet and Health-Related Quality of Life in University Students. Nutrients.

[40] Seral-Cortes M., Sabroso-Lasa S., Bailo-Aysa A., Gonzalez-Gross M., Molnar D., Censi L., Molina-Hidalgo C., Gottrand F., De Henauw S., Manios Y. (2021). Mediterranean Diet, screen-time-based sedentary behavior and their interaction effect on adi-posity in European adolescents: The HELENA Study. Nutrients.

[41] Aljefree N., Ahmed F. (2015). Association between dietary pattern and risk of cardiovascular disease among adults in the Middle East and North Africa region: A systematic review. Food Nutr. Res..

[42] Kilcast D. (2010). Sensory Analysis for Food and Beverage Quality Control.

[43] United Nations Statistics Division (2017). National Accounts main Aggregates Database. https://unstats.un.org/unsd/snaama/.

[44] United Nations Development Programme (2019). Human Development Indices and Indicators: 2019 Statistical Update.

[45] Naja F., Nasreddine L., Itani L., Chamieh M.C., Adra N., Sibai A.M., Hwalla N. (2011). Dietary patterns and their association with obesity and sociodemographic factors in a national sample of Lebanese adults. Public Health Nutr..

